# CHA zeolite prepared using caesium hydroxide as a single mineralising agent

**DOI:** 10.1039/d6cc04556a

**Published:** 2026-09-11

**Authors:** Yongmei Guo, Robert S. Tapping, Pacôme Chapel, Chetna Tyagi, Hayley G. Andrews, Svetlana Mintova, Lubomira Tosheva

**Affiliations:** a Faculty of Science and Engineering, Manchester Metropolitan University Chester Street Manchester M1 5GD UK l.tosheva@mmu.ac.uk; b Minjiang University, Fujian Key Laboratory of Novel Functional Textile Fibers and Materials Fuzhou 350108 Fujian China; c Normandie Université, ENSICAEN, UNICAEN, CNRS, Laboratoire Catalyse et Spectrochimie (LCS) 14000 Caen France

## Abstract

We report the organic template-free synthesis of zeolite CHA using caesium hydroxide as a single mineralising agent. The Cs-CHA zeolites obtained have unusual textural porosity and higher yields compared to Na,Cs-CHA zeolites prepared with the addition of both sodium hydroxide and caesium hydroxide.

## Abstract 2

Zeolite Cs-CHA was prepared in the presence of CsOH as a single mineralising agent. Compared to Na,Cs-CHA made with both CsOH and NaOH, the Cs-CHA zeolites reported displayed unusual textural porosity and higher yields.

## Graphical abstract

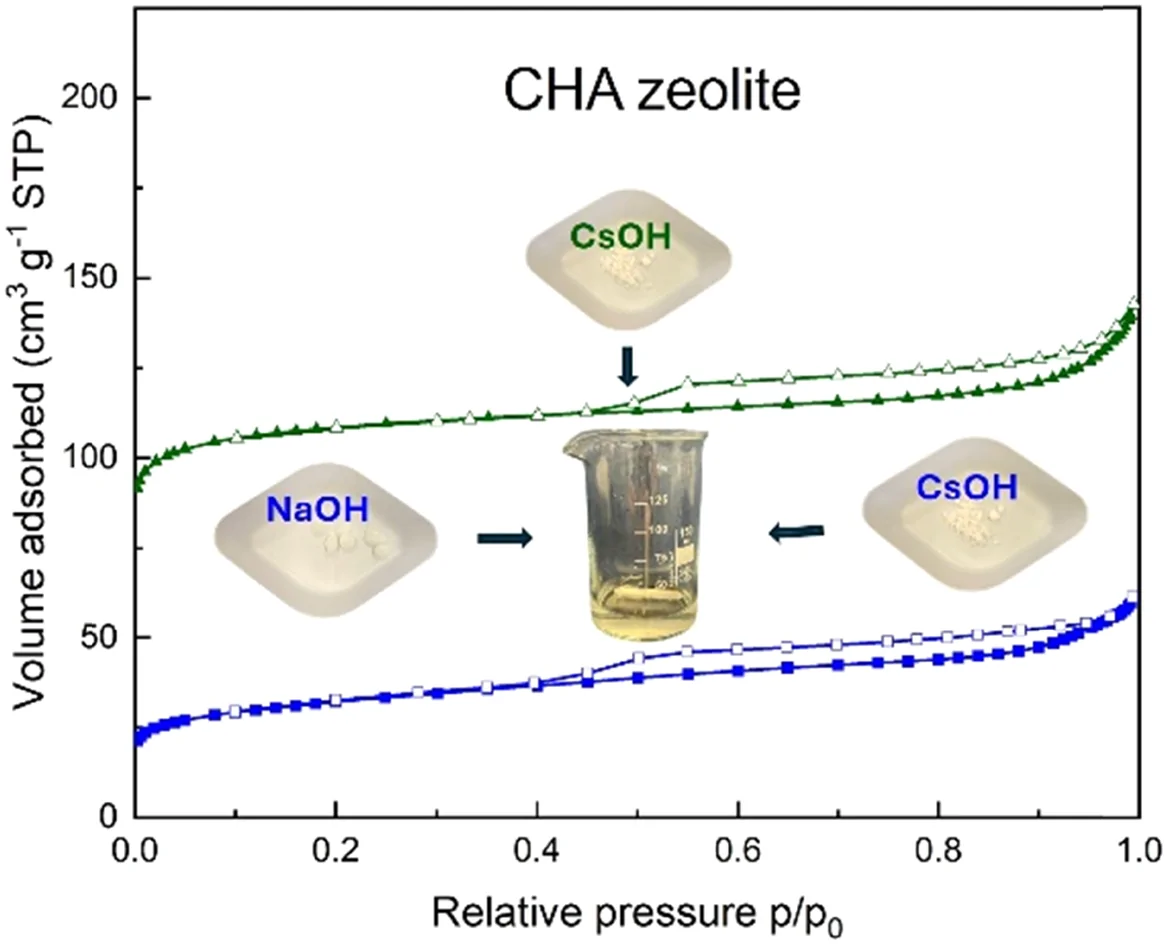

Zeolites are crystalline aluminosilicate materials with widespread applications in adsorption, separation, ion-exchange and catalysis.^1,2^ Organic structure-directing agents (OSDAs) are often used in the zeolite synthesis to make new structures or to tune their composition, morphology and structural defects. However, the use of OSDAs requires post-synthesis calcination to open the zeolite porous structure, which increases the energy consumption and the CO_2_ footprint. Therefore, the synthesis of zeolites from organic template-free systems is highly desirable.^3^

Among different zeolites, the small-pore CHA zeolite is one of the most-studied zeolites due to its application in gas separations (low-silica CHA)^4,5^ and catalysis for the selective reduction of NOx and methanol-to-olefins (high-silica SSZ and SAPO-34).^6^ The CHA-type framework consists of double six-membered rings (DR6) and large *cha* cages (*ca.* 6.7 Å × 10 Å), which are accessible through 3.8 Å × 3.8 Å eight-membered ring (8MR) openings.^7^ Extra-framework balancing cations may coordinate in the 8MRs of the CHA-type zeolite, and when all 8MRs are saturated with such cations, which is achieved at Si/Al < 3, a “trapdoor” adsorption mechanism is observed.^8,9^ In this mechanism, the gate-keeping cations deviate from their position in the 8MRs to allow “strong” components such as CO_2_ to enter the *cha* cages, while “weak” components such as CH_4_ and N_2_ are blocked.

Different strategies have been applied to synthesise low-silica CHA zeolites with reduced use, or in the absence of OSDAs. Examples include synthesis in fluoride media,^10,11^ hydrothermal conversion of FAU (Y) zeolite in the presence of KOH,^12^ or seed-assisted methods.^13–18^ The preparation of CHA-type zeolite in the presence of alkali hydroxides as mineralising agents has gained interest in recent years because of the OSDA-free one-pot nature of the synthesis, often at temperatures below 100 °C, that does not require the use of hazardous autoclaves.^5,19–23^ Typically, the as-made CHA zeolites show negligible nitrogen adsorption because the extra-framework cations such as Cs^+^ sit in the 8MRs and do not allow the N_2_ to enter the *cha* cages.^24^

Recently, we reported the synthesis of Na,Cs-CHA zeolite with a pompom morphology in the presence of Na^+^ and Cs^+^ as dual mineralising and structure directing agents.^25^ Herein, we expand on our previous work and explore the synthesis of CHA zeolite (Cs-CHA) in the presence of Cs^+^ as a single mineralising agent and compare its characteristics with those of Na,Cs-zeolite prepared by tandem Na^+^–Cs^+^ templating. Sodium aluminate was used as an alumina source in the synthesis of both zeolites; NaOH was added too for the synthesis of Na,Cs-CHA but not for Cs-CHA. The weight of all purified zeolite samples prepared exceeded 2.4 g, which, considering that the weight of (SiO_2_ + Al_2_O_3_) added to the precursor gel was 1.92 g, indicated very high zeolite yields. In addition, the weight of the Cs-CHA zeolites was about 15% higher than that of Na,Cs-CHA, indicating higher yields for samples prepared with just CsOH. The most striking difference between Na,Cs-CHA and Cs-CHA samples was the unusual increase in the nitrogen adsorption of the latter, with BET surface areas and micropore volumes increasing from *ca.* 110 m^2^ g^−1^ and 0.03 cm^3^ g^−1^ to up to *ca.* 400 m^2^ g^−1^ and 0.16 cm^3^ g^−1^, respectively. This work is the first example of an *in situ* control of the accessibility of the pore structure of zeolite CHA to nitrogen, which could open new application avenues for zeolite CHA in gas adsorption and separation, and could possibly be translated into other small-pore zeolites.

The crystallisation of zeolite CHA in the presence of 0.5 g of CsOH was followed by XRD, XRF, Raman, and nitrogen adsorption measurements, and the results were compared to those of Na,Cs-CHA prepared with the addition of 0.5 g of CsOH and 0.91 g of NaOH. Fig. 1(a) indicates that the crystallisation in the presence of Cs as a single mineralising agent took 5 days compared to 1 day for Na,Cs-CHA. Extending the hydrothermal treatment of the latter sample to 5 days resulted in CHA/ANA intergrown zeolites in agreement with other reports in the literature (Fig. S1).^21^ The sample prepared in exactly the same way as Na,Cs-CHA but without the addition of Cs resulted in a FAU-type zeolite (Fig. S2). This indicates that the presence of Cs is essential for the formation of zeolite CHA under the conditions used here. The XRF data presented in Table S1 show that both the Cs and the Al content increase with increasing hydrothermal treatment time from 3 to 5 days while keeping the Cs/Al ratio constant, which could explain the longer time required for the synthesis of crystalline zeolite CHA when using Cs as a single mineralising agent.

**Fig. 1:**
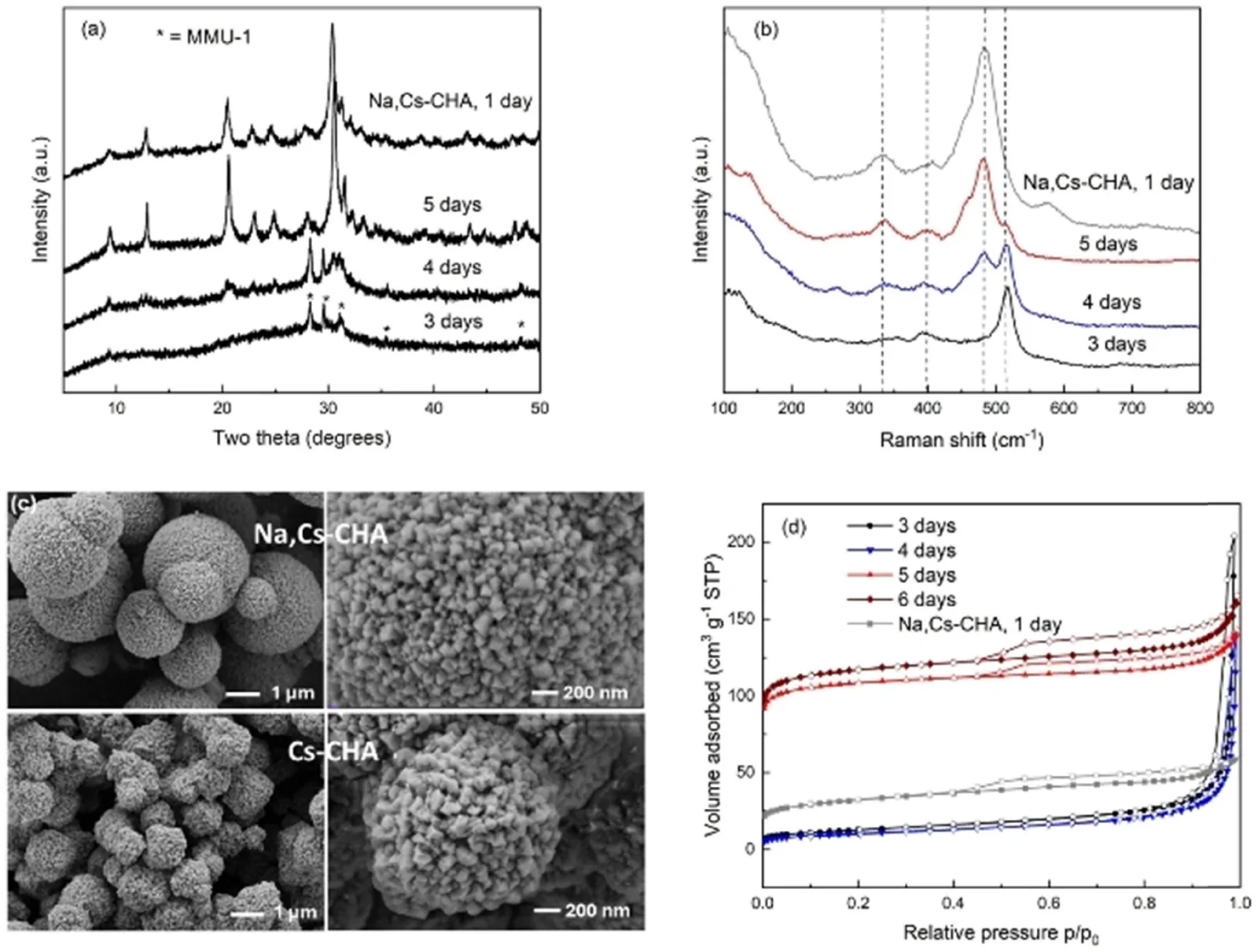
(a) XRD patterns, (b) Raman spectra, (c) SEM images at two different magnifications, and (d) nitrogen adsorption–desorption isotherms at −196 °C (no displacement along the *y*-axis) of Cs-CHA samples prepared with 0.5 g of CsOH at different treatment times and the Na,Cs-CHA reference zeolite.

The samples prepared after 3 and 4 days of treatment contained a mixture of amorphous material and zeolite MMU-1.^26^ The Si/Al ratio of Na,Cs-CHA (1.67) was lower compared to the Si/Al ratios of Cs-CHA zeolites (∼2.2) as a result of the higher cation content/higher alkalinity of the Na,Cs-CHA precursor gel.^13,27^ Raman analysis further confirmed the XRD results (Fig. 1(b)). The 3 day sample exhibited a peak at 517 cm^−1^ corresponding to the 4MRs of MMU-1.^26^ After 4 days of hydrothermal treatment, both the MMU-1 band at 517 cm^−1^ and the CHA band at 483 cm^−1^ were observed in the corresponding Raman spectrum, and for the 5 day and the Na,Cs-CHA samples, the band at 483 cm^−1^ was dominant. The intensity of the band at 335 cm^−1^, which is associated with 6MRs, increased with an increase in the time of treatment, which could be linked to the formation of DR6 in CHA.^14^

Both Na,Cs-CHA and Cs-CHA samples were built of similar 50–100 nm crystals, which were arranged into larger aggregates of different morphologies (Fig. 1(c)). The Na,Cs-CHA contained pompom-like spherical particles with a dual particle-size distribution, with sizes of around 1 µm and 3 µm, respectively. The particle size distribution of the aggregates in the Cs-CHA sample was more uniform, around 1.5 µm, with an irregular spherical morphology. The nanosized nature of the CHA crystals is expected to be beneficial for gas separation applications,^28^ whereas the nanocrystal arrangement into micron-sized spherical aggregates makes the sample purification step more energy efficient than that of colloidal nanozeolites.

The most striking difference between the two samples was observed in the nitrogen adsorption isotherms (Fig. 1(d)). The 3 day and 4 day samples showed type II adsorption isotherms with negligible adsorption of nitrogen. The total pore volume decreased, from 3 to 4 days of treatment, from 0.31 to 0.21 cm^3^ g^−1^ and levelled at about 0.22–0.26 cm^3^ g^−1^ for the crystalline Cs-CHA samples (Table S1). The isotherms of Na,Cs-CHA and Cs-CHA samples exhibited similar shapes, with a steep increase in the volume adsorbed at low relative pressures and a hysteresis loop at *p*/*p*_0_ of 0.4 or 0.45, respectively, both of which were enhanced for the Cs-CHA samples. The textural data show a substantial increase in the BET surface areas (∼3.5 times from *ca.* 113 m^2^ g^−1^ to *ca.* 350–380 m^2^ g^−1^) and micropore volumes (∼5 times from 0.028 cm^3^ g^−1^ to about 0.14 cm^3^ g^−1^). The increase in the hydrothermal treatment from 5 to 6 days resulted in a slight increase in the BET surface area and micropore volume of Cs-CHA. Our attempt to upscale the Cs-CHA sample four times showed that after 5 days of hydrothermal treatment, MMU-1 impurities were still present in the product (Fig. S3). The hydrothermal treatment for the upscaled sample was increased to 7 days to obtain the Cs-CHA sample with the same characteristics (Fig. S3 and Table S1). These differences were more pronounced compared to our previous results for the upscaling of the CHA zeolite prepared by Na–Cs templating.^25^ The morphology of all crystalline Cs-CHA samples prepared with 0.5 g of CsOH was similar (Fig. S4).

We also explored the possibility of further decreasing the amount of CsOH added to make Cs-CHA zeolites, and we performed a series of experiments using 0.3 g of CsOH (Fig. 2). The zeolite crystallisation was considerably slower, and it took 14 days to prepare Cs-CHA of similar crystallinity to the crystallinity of the 0.5 g samples, albeit the sample contained minor LTA impurities (Fig. 2(a)). The sample after 7 days of treatment adsorbed a negligible amount of nitrogen and had a higher pore volume and a higher Si/Al ratio compared to the samples obtained after 10 and 14 days (Table S2 and Fig. 2(b)). After 10 days of treatment, the sample's textural characteristics were already similar to that of the 0.5 g CsOH Cs-CHA samples. The BET surface area of the 14 day sample increased to *ca.* 400 m^2^ g^−1^, and the micropore volume increased to 0.16 cm^3^ g^−1^. The SEM analysis of the 10 day and 14 day Cs-CHA samples indicated that both samples displayed similar particle size distributions and macroscopic shape of the large aggregates (Fig. 2(c) and (d)). However, the primary nanocrystals appeared smaller in the 10 day sample and exhibited a slightly different arrangement within the aggregates compared with those in the 14 day sample (the insets in Fig. 2(c) and (d)).

**Fig. 2:**
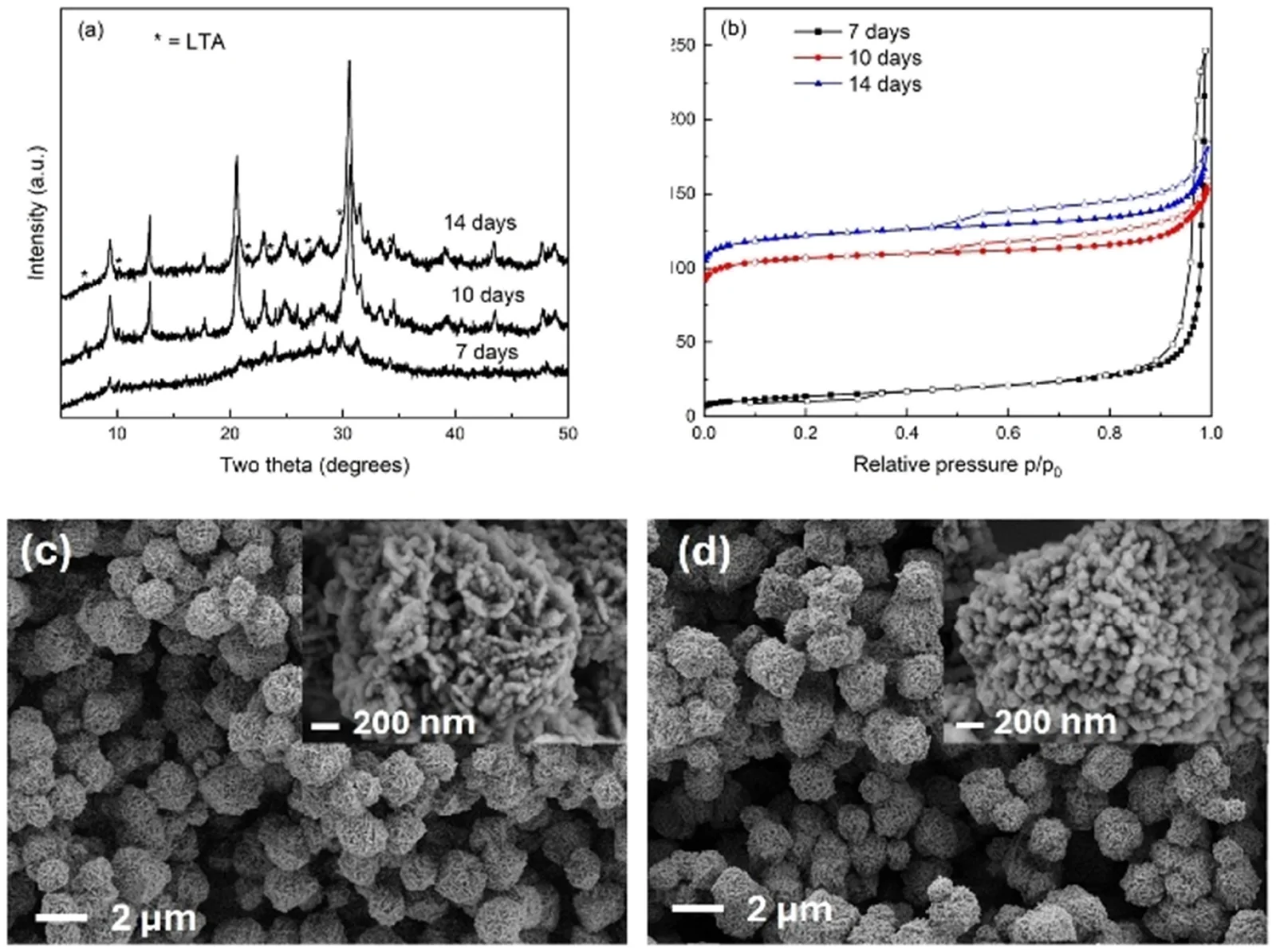
(a) XRD patterns, (b) nitrogen adsorption–desorption isotherms, and SEM images of (c) Cs-CHA prepared after 10 days of hydrothermal treatment and (d) Cs-CHA prepared after 14 days of hydrothermal treatment; the Cs-CHA samples were prepared with 0.3 g of CsOH.

To gain insights into the role of Cs as a structure-directing agent, we compared the features of the Cs-CHA zeolite prepared with different amounts of CsOH (1.18 g, 0.59 g, 0.5 g and 0.3 g) (Table S3). The Cs content decreased with a decrease in the amount of CsOH added, and it was balanced out by Na. The 1.18 g sample showed that the amounts of Cs and Na were approximately the same, and the sample had a lower Si/Al ratio compared to the other Cs-CHA samples. This sample was pure ANA zeolite with a pomegranate-like morphology containing 0.7–1.1 µm crystals (Fig. S5). The other three samples were zeolite CHA with similar pore structures (Fig. S6). Their morphology was also similar, although the 0.3 g sample displayed somewhat larger aggregates built of smaller nanoparticles compared to the 0.59 g and 5 g samples (Fig. S7).

Powder XRD patterns of the 0.59 g, 0.5 g, and 0.3 g of Cs-CHA and Na,Cs-CHA samples collected for Le Bail refinement are shown in Fig. S8. All diffraction patterns were refined assuming the trigonal space group *R*3̄*m* (hexagonal setting), as is commonly reported for CHA-type zeolites.^7^ The Na,Cs-CHA sample contained minor FAU impurities. Its XRD pattern displayed broader peaks, especially for the non-zero *l* planes (as highlighted by the (101) reflection and the complete disappearance of the (003) reflection). Assuming similar crystallite morphologies for both types of compounds (Fig. 1(c)), these features are attributed to anisotropic micro-strain effects occurring preferentially along the *c*-axis. In addition, there is an unassigned peak at ∼8° two theta, which could be associated with a small amount of stacking faults in ABC-D6R zeolites.^29^ Such stacking faults are known to occur preferentially along the *c*-axis, which is also consistent with the preferential broadening of reflections with non-zero *l* indices. Moreover, there was a slight shift toward lower two theta angles compared to the Cs-CHA samples. The diffraction patterns of the Cs-CHA samples were similar to each other, with a slight shift toward lower two theta angles with increasing Cs content. The relative crystallinity of the Cs-CHA samples was similar, whereas the Na,Cs-CHA showed slightly lower crystallinity (Fig. S9).

The Le Bail refinement of the 0.5 g Cs-CHA sample and the Na,Cs-CHA sample is shown in Fig. S10, and a summary of the refinement data for all samples is provided in Table S4. The changes in the unit cell parameters as a function of the Cs and Na atoms per unit cell are illustrated in Fig. 3. The (Cs + Na) content of the three Cs-CHA samples is similar, and as Cs decreases the amount of Na increases. The unit cell expands with an increase in the Cs content, which is expected considering the larger size of Cs^+^.^30^ The larger unit cell of Na,Cs-CHA can be linked to the higher Cs and (Na + Cs) content and to the lower Si/Al ratio of this sample compared to Cs-CHA samples.^31^

**Fig. 3:**
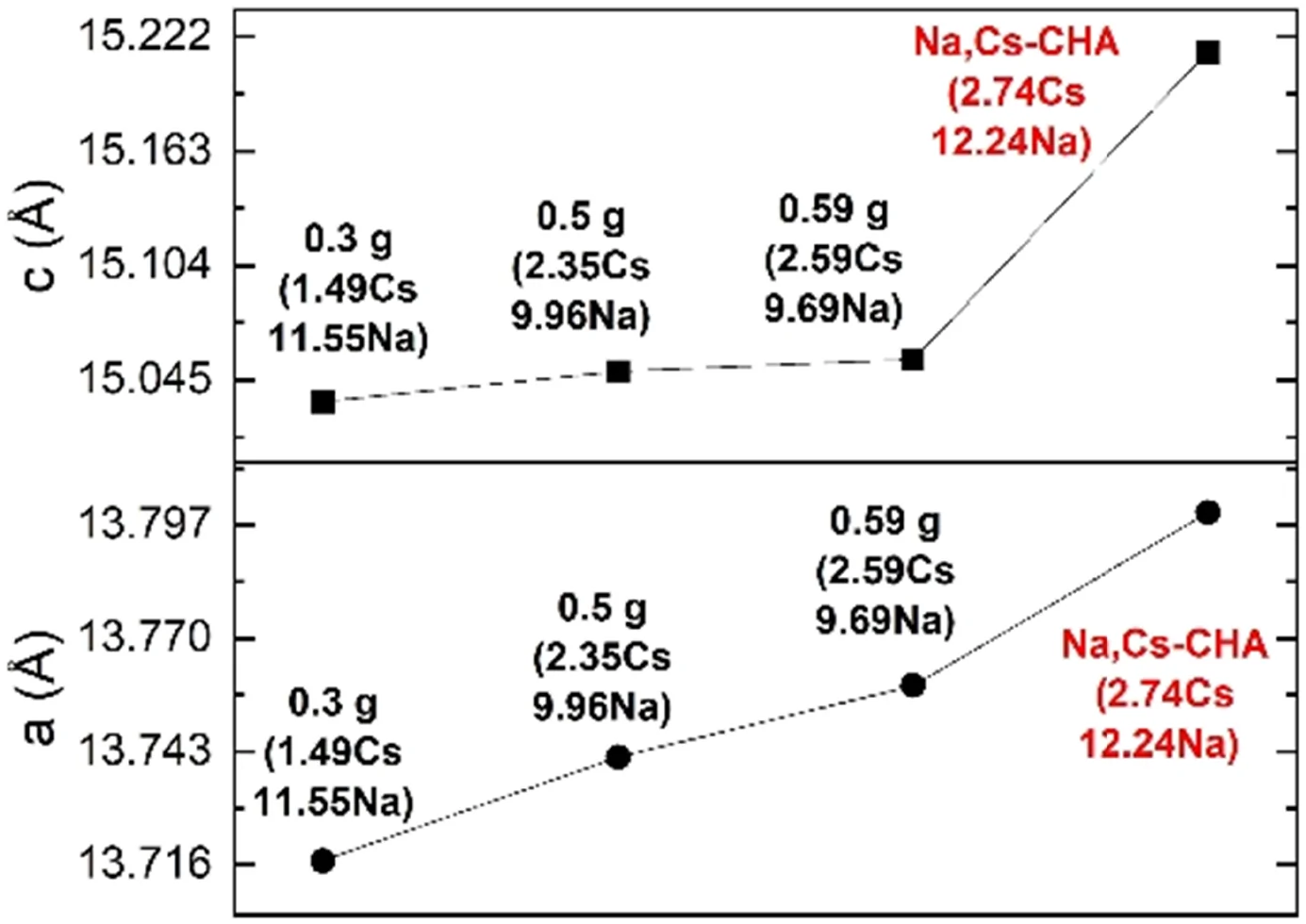
Variations in the unit cell parameters for Cs-CHA samples prepared with different amounts of CsOH added and the reference Na,Cs-CHA sample. The number of Na and Cs atoms per unit cell is provided in brackets.

The focus of this work was to explore the zeolite CHA synthesis in the presence of CsOH. Considering the substantial differences in the Si/Al ratio between the Cs-CHA and Na,Cs-CHA samples, we have further attempted to prepare Na,Cs-CHA zeolites with comparable Si/Al ratios to the Cs-CHA samples. Our previous results have shown that all Na,Cs-CHA samples prepared using 0.45–0.91 g of NaOH and varying amounts of CsOH (0.3–0.7 g) had Si/Al ratios of about 1.7 and BET surface areas around 100 m^2^ g^−1^. Therefore, we have now performed additional syntheses using less NaOH (0.2 g or 0.35 g) and a constant amount of CsOH (0.7 g) (Table S5 and Fig. S11). These experiments confirm that CsOH has a structure-directing role in the crystallisation of zeolite CHA, whereas the NaOH added accelerates the zeolite CHA formation. Decreasing the amount of NaOH from 0.35 g to 0.2 g slowed down the crystallisation and the hydrothermal time had to be increased to 2 days for the latter sample to obtain a highly crystalline product. The solid yield of these two Na,Cs-CHA samples was still about 15% lower compared to the yield of Cs-CHA samples. Increasing the Si/Al ratio of Na,Cs-CHA resulted in higher BET surface areas and micropore volumes. This indicates that the differences in the textural porosity of Na,Cs-CHA and Cs-CHA (Fig. 1d) could be due to differences in the zeolite framework composition and framework charge density. Nevertheless, the isotherms of Cs-CHA and Na,Cs-CHA (Si/Al = 2.06) show differences at relative pressures of >0.5 (Fig. S11a), and further tests, such as Rietveld analysis, to determine the positions of Cs^+^ and Na^+^ in the Cs-CHA and Na,Cs-CHA structures are required to explain these differences.

In summary, we have reported the high-yield OSDA-free synthesis of zeolite CHA in the presence of caesium hydroxide as a single mineralising agent. Compared to the Na,Cs-CHA zeolite made with both NaOH and CsOH, the Cs-CHA zeolites show an unusual increase in the adsorption of nitrogen. Such *in situ* control over the accessibility of nitrogen into the pores of the low-silica CHA zeolite and control over the relative distribution of Na and Cs extra-framework cations is reported for the first time. These findings may expand the application of zeolite CHA in gas adsorption and separation, and could be applied to the synthesis of other small-pore zeolites.

## Author contributions

Y. Guo: investigation, vizualization, and validation. R. S. Tapping: investigation, vizualization, and formal analysis. P. Chapel: formal analysis, vizualization, and writing – review & editing. C. Tyagi: investigation. H. G. Andrews: investigation. S. Mintova: conceptualization and writing – review & editing. L. Tosheva: conceptualization, project administration, supervision, writing – original draft, and writing – review & editing.

## Conflicts of interest

There are no conflicts to declare.

## Supplementary Material

CC-062-D6CC04556A-s001

## Data Availability

The data supporting this article have been included as part of the supplementary information (SI). Supplementary information: experimental procedures and supplementary sample characterisation data. See DOI: https://doi.org/10.1039/d6cc04556a.
