# Supplementary material for: CHA zeolite prepared using caesium hydroxide as a single mineralising agent
Source: Chem Commun (Camb). 2026 Sep 11;62(81):20356–9. doi: 10.1039/d6cc04556a (PMC13596086; doi:10.1039/d6cc04556a)
Supplement: Supplementary file 1 [file CC-062-D6CC04556A-s001.pdf]

Supplementary Information (SI) for ChemComm.
This journal is © The Royal Society of Chemistry 2026

Electronic Supplementary Information

CHA zeolite prepared using caesium hydroxide as a single mineralising agent

Yongmei Guo,[a,b] Robert S. Tapping,[a] Chetna Tyagi,[a] Hayley G. Andrews,[a] Pacôme Chapel,[c] Svetlana Mintova,[c] and Lubomira Tosheva*[a]

[a] Faculty of Science and Engineering, Manchester Metropolitan University, Chester Street, Manchester M1 5GD, United Kingdom.

[b] Minjiang University, Fujian Key Laboratory of Novel Functional Textile Fibers and Materials, Fuzhou 350108, Fujian, China.

[c] Normandie Université, ENSICAEN, UNICAEN, CNRS, Laboratoire Catalyse et Spectrochimie (LCS), 14000 Caen, France.

*Corresponding author: Lubomira Tosheva (l.tosheva@mmu.ac.uk)

Synthesis of Na,Cs-CHA and Cs-CHA zeolites

Na,Cs-CHA zeolite was prepared from a gel with the molar composition 10.0SiO_2 : 2.22Al_2O_3 : 8.13Na_2O : 0.58Cs_2O : 381H_2O. The precursor gels were prepared by dissolving 1.03 g sodium aluminate ($NaAlO_2$, 50.5% Al_2O_3, 44.1% Na_2O, Fisher Scientific) in 12.42 g distilled water in a beaker under stirring. The beaker was then placed in a large Petri dish with ice water to keep the temperature constant upon the addition of sodium hydroxide (NaOH pellets, analytical reagent grade, Fisher Scientific) (0.91 g) and caesium hydroxide hydrate ($CsOH \cdot xH_2O$, 99.9%, 15−20% H_2O, Alfa Aesar; 20% water content was used for the calculations of the molar compositions provided) (0.5 g). After complete dissolution of the hydroxides, the ice bath was removed, 4.66 g of colloidal silica (Ludox HS-30, Aldrich) were added dropwise, and the mixture was stirred for 2 hours. After the stirring, the gel was transferred to 125 mL polypropylene reactor and hydrothermally treated at 80 °C for 24 h. The zeolite obtained were purified by three-times centrifugation and redispersion in distilled water and dried at 80 °C overnight. Finally, the sample was grinded and stored in a glass vial at ambient conditions.

The Cs-CHA zeolites were prepared in a similar way without the addition of NaOH. The amount of CsOH added was varied (0.3 g, 0.5 g, 0.59 g, 1.18 g) and the hydrothermal treatment at 80 °C was adjusted accordingly between 5 days and 2 weeks. The gel molar compositions for the Cs-CHA zeolites were in the range 10.0SiO_2 : 2.22Al_2O_3 : 3.17Na_2O : (0.34-1.37)Cs_2O : (380-384)H_2O for the 0.3 g – 1.18 g of CsOH used, respectively. The sample prepared with 0.5 g was also upscaled four-times with all initial reagents multiplied by 4 (e.g., 4.12 g of $NaAlO_2$, etc.) and the hydrothermal treatment performed in a 500 mL polypropylene reactor.

Characterisation methods

Crystalline phases were determined from X-ray diffraction (XRD) patterns collected with a PANalytical X'pert Powder X-ray diffractometer using Cu $K\alpha_1$ and $K\alpha_2$ radiation (λ = 1.5406 and 1.5444 Å respectively) and a PIXCel 1-D detector. Data were collected in the range 5 -50° 2θ with a step size of 0.013° 2θ and a measuring time of 89 s per step. The samples were rotated at 60 rpm during the data collection. Automatic divergence and antiscatter slits were used on the incident and diffracted beam paths. The morphology of the zeolites prepared was studied with a scanning electron microscope (SEM) (Carl Zeiss Supra 40 V). Samples were sputtered with gold prior to analysis. The chemical composition of the samples was determined using an X-ray fluorescence (XRF) instrument (Rigaku NEX-CG energy-dispersive X-ray fluorescence spectrometer). Nitrogen adsorption isotherm measurements at −196 °C were performed with a Micrometrics ASAP 2020 surface area analyser. Samples were degassed at 300 °C overnight prior to analysis. The Brunauer–Emmett–Teller (BET) method was used to calculate the samples' surface areas, and Barrett–Joyner–Halenda (BJH) pore-size distributions were determined from the desorption branch of the isotherms. Micropore volumes and external surface areas were determined by the t-plot method, whereas total pore volumes were calculated from the volume adsorbed at a relative pressure of 0.995. Raman spectra were collected from five different areas across each zeolite sample using a Horiba Scientific XploRA Plus Raman Microscope. Measurements were made using a 638 nm laser fitted with a 100x objective, a 10 0μm confocal hole, a 100 μm slit and a 1200 gr/mm diffraction grating. Spectra were collected with a 50s acquisition time for 5 accumulations.

Powder X-ray diffraction (PXRD) patterns for the Le Bail refinement were collected on an Anton Paar XRDynamic 500 diffractometer equipped with a graphite/Ni monochromator in Bragg–Brentano geometry, using Cu $K\alpha_1$ and $K\alpha_2$ radiation (λ = 1.5406 and 1.5444 Å, respectively; 40 kV, 50 mA) and a Pixos 2000 detector. Diffraction patterns were recorded

over the 2θ range of 5–90 ° with a step size of 0.01° and a counting time of 1204.15 s per step with fixed divergence slits. Le Bail profile refinements were performed using the FullProf Suite software to determine the unit-cell parameters and volumes. Instrumental broadening parameters were determined by refining a LaB_6 reference material. Peak broadening was subsequently modeled by considering contributions from both crystallite size and microstrain effects.

Supplementary figures and tables:

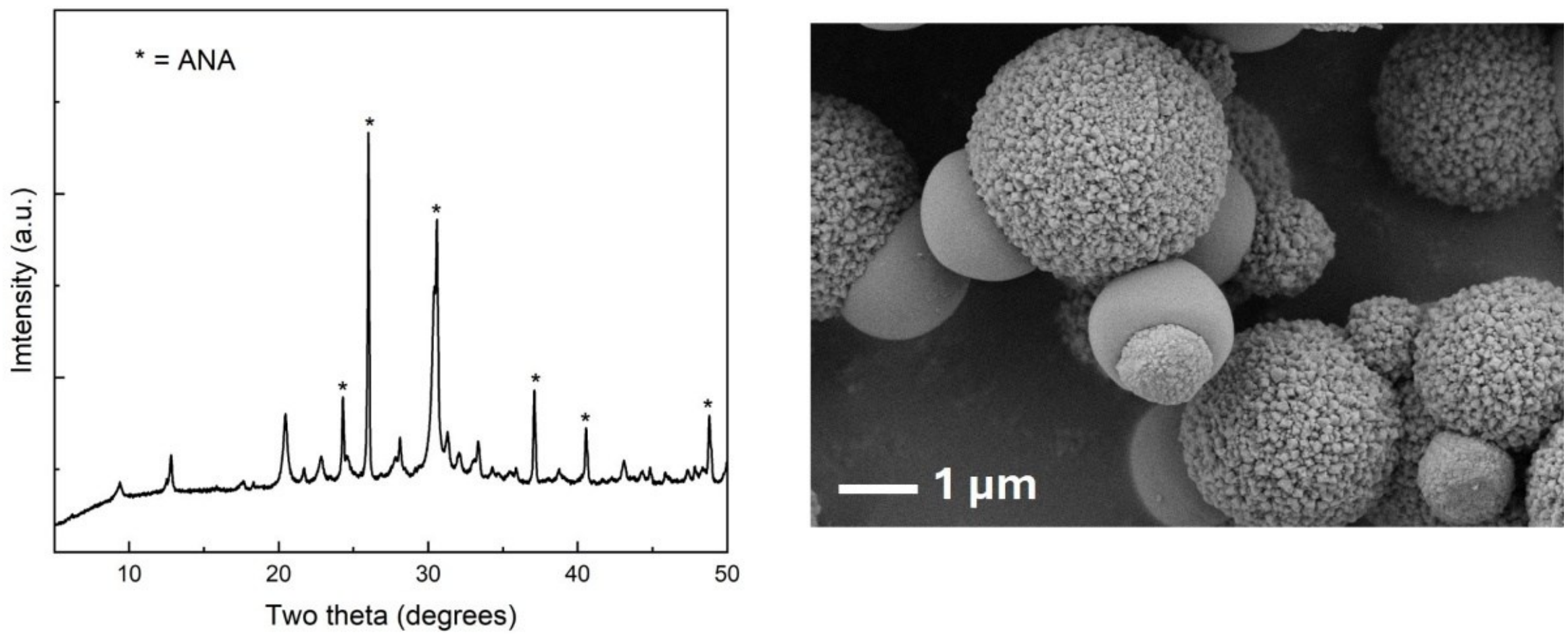

Figure S1. XRD pattern (left) and SEM image (right) of the Na,Cs-CHA sample prepared with 0.5 g CsOH for 5 days of hydrothermal treatment indicating the co-presence of ANA.

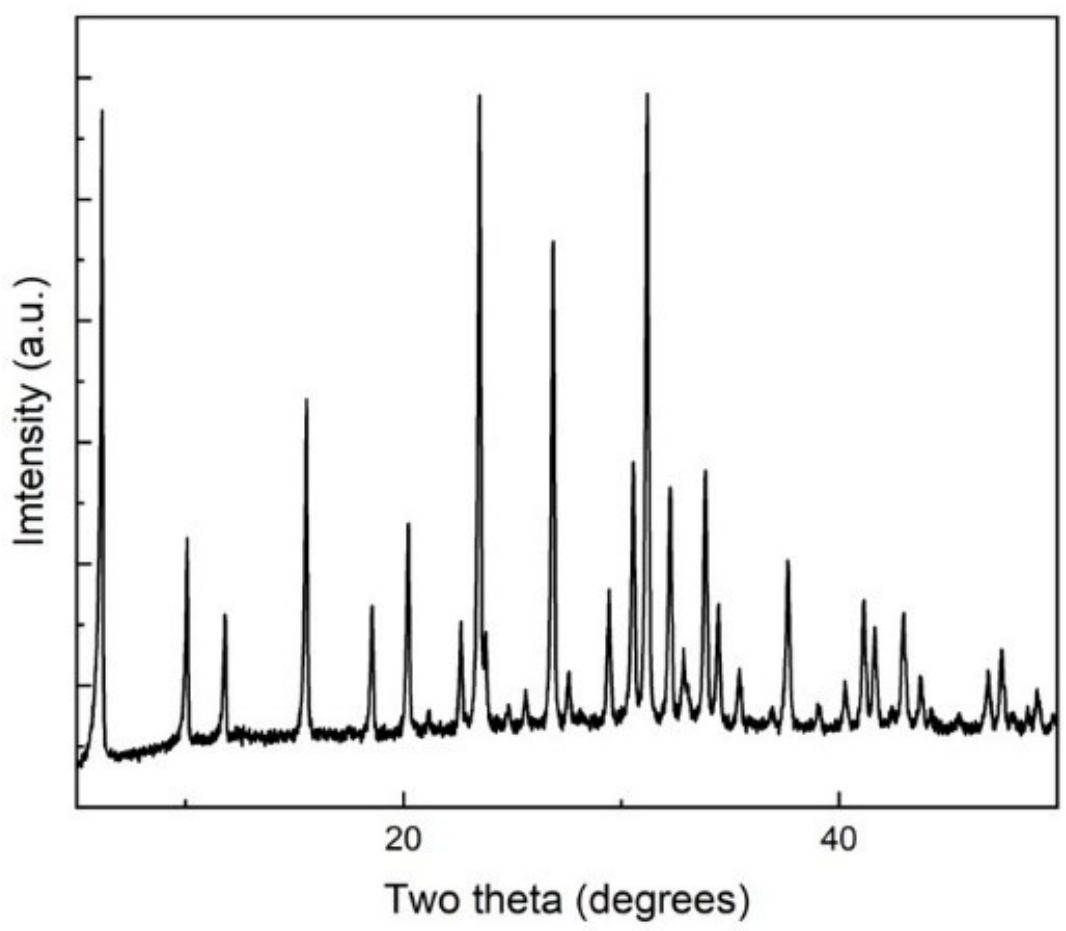

Figure S2. XRD pattern of FAU sample prepared in exactly the same way as Na,Cs-CHA but without the addition of Cs.

Table S1. Chemical composition (mol. ratios) and textural characteristics of Cs-CHA samples prepared with 0.5 g CsOH at different hydrothermal treatment times and the Na,Cs-CHA zeolite.

Sample	Si/Al	Cs/Al	Na/Al	S_{BET} (m^2 g^{-1})	S_{EXT} (m^2 g^{-1})	V_{micro} (cm^3 g^{-1})	V_{total} (cm^3 g^{-1})
Cs-CHA*, 3 days	2.60	0.23	1.00	45.5	41.8	0.001	0.31
Cs-CHA*, 4 days	2.28	0.23	1.06	37.2	35.6	-	0.21
Cs-CHA, 5 days	2.17	0.22	0.92	352.1	66.1	0.138	0.22
Cs-CHA, 6 days	2.19	0.21	0.91	381.4	83.1	0.145	0.26
Cs-CHA, 7 days (upscaled)	2.19	0.21	0.88	368.4	75.7	0.142	0.25
Na,Cs-CHA, 1 day	1.67	0.20	0.91	113.7	56.3	0.028	0.09
Na,Cs-CHA*, 5 days	1.71	0.21	0.92	82.4	51.2	0.015	0.01
FAU, 1 day**	1.66	-	1.17	710.9	45.2	0.316	0.36

*- the samples are not pure CHA zeolites, see text for further details.
n.m. – not measured
** - FAU sample prepared in exactly the same way as Na,Cs-CHA but without the addition of CsOH

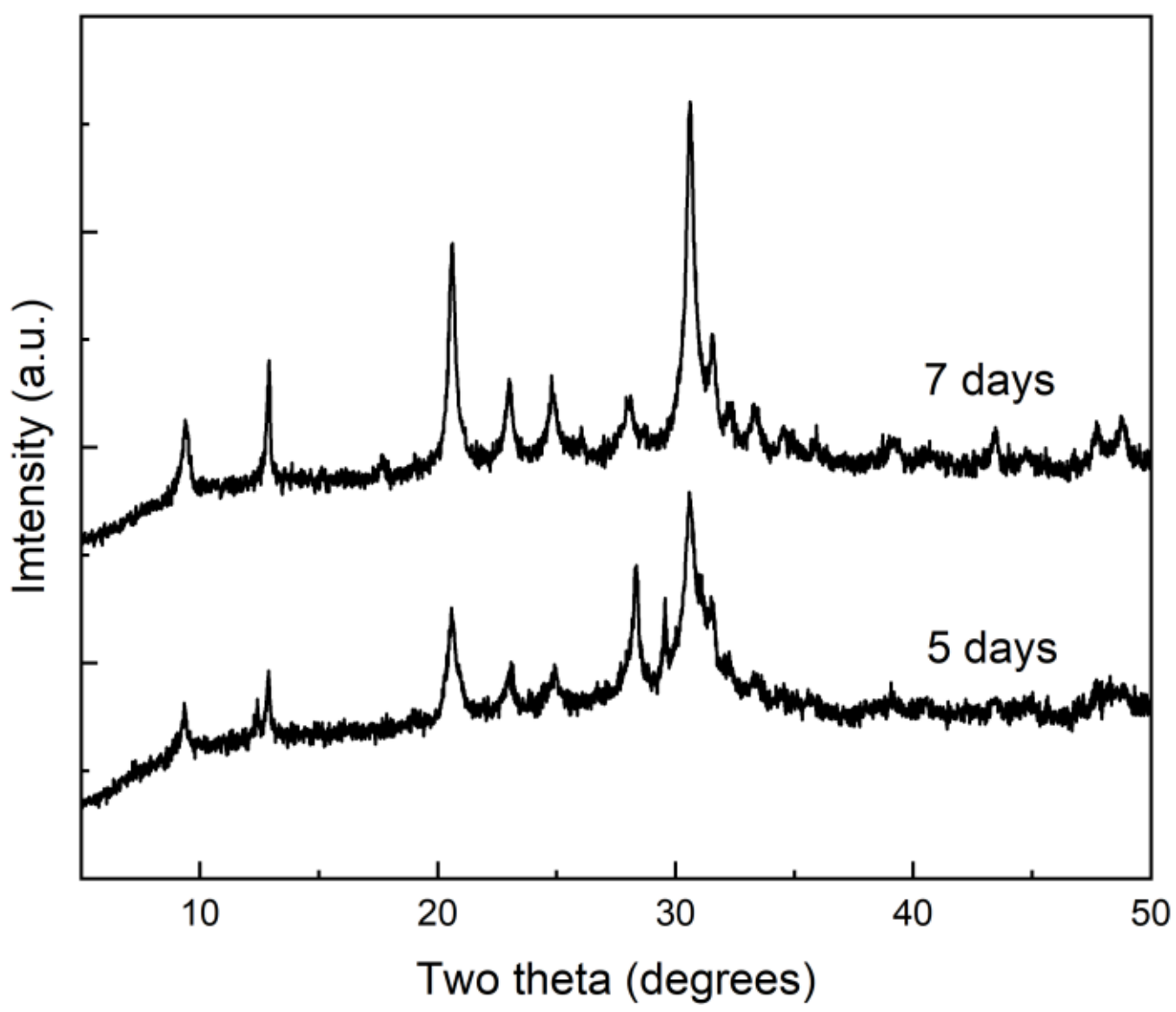

Figure S3. XRD patterns of the Cs-CHA sample prepared with 0.5 g CsOH from the four-times scaled-up synthesis after 5 and 7 days of hydrothermal treatment.

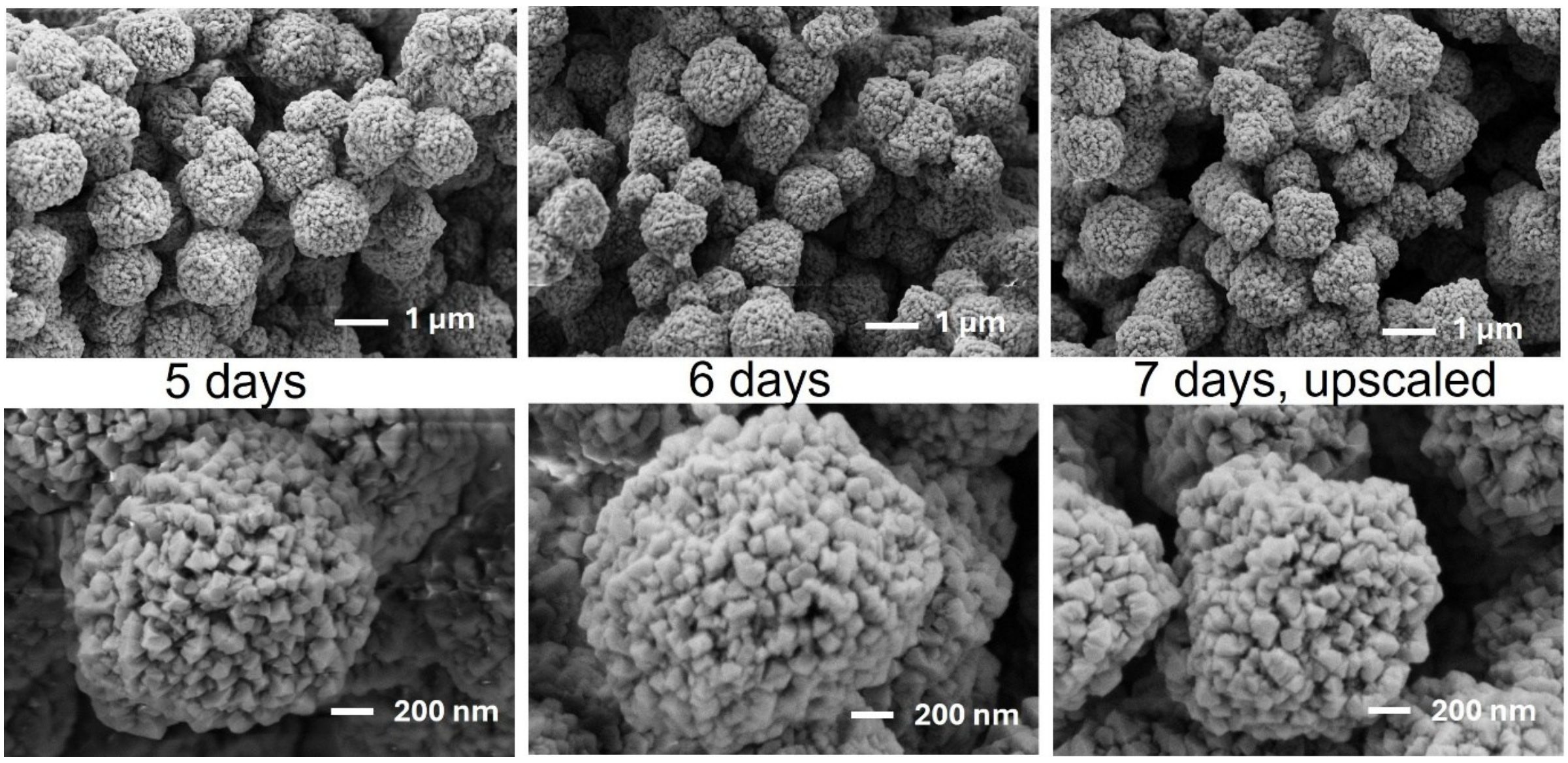

Figure S4. SEM images of Cs-CHA samples prepared with 0.5 g CsOH.

Table S2. Chemical composition (mol. ratios) and textural characteristics of Cs-CHA samples prepared with 0.3 g CsOH at different hydrothermal treatment times.

Sample	Si/Al	Cs/Al	Na/Al	S_{BET} (m^2 g^{-1})	S_{EXT} (m^2 g^{-1})	V_{micro} (cm^3 g^{-1})	V_{total} (cm^3 g^{-1})
Cs-CHA*, 7 days	2.48	0.12	1.08	48.6	47.01	-	0.38
Cs-CHA, 10 days	2.11	0.12	1.02	346.7	58.1	0.140	0.25
Cs-CHA, 14 days	2.21	0.13	1.03	396.6	75.8	0.156	0.28

*- the sample is not pure CHA zeolite.

Table S3. Chemical composition (mol. ratios) and textural characteristics of Cs-CHA samples prepared with different amounts of CsOH.

Sample	Si/Al	Cs/Al	Na/Al	S_{BET} (m^2 g^{-1})	S_{EXT} (m^2 g^{-1})	V_{micro} (cm^3 g^{-1})	V_{total} (cm^3 g^{-1})
1.18 g, 4 days	1.99	0.61	0.62	n.d.	n.d.	n.d.	n.d.
0.59 g, 5 days	2.23	0.23	0.87	365.6	78.6	0.139	0.23
0.5 g, 7 days (upscaled)	2.19	0.21	0.88	368.4	75.7	0.142	0.25
0.3 g,14 days	2.21	0.13	1.03	396.6	75.8	0.156	0.28

n.d. – not determined; very low nitrogen uptake (BET surface area < 1 m^2 g^{-1})

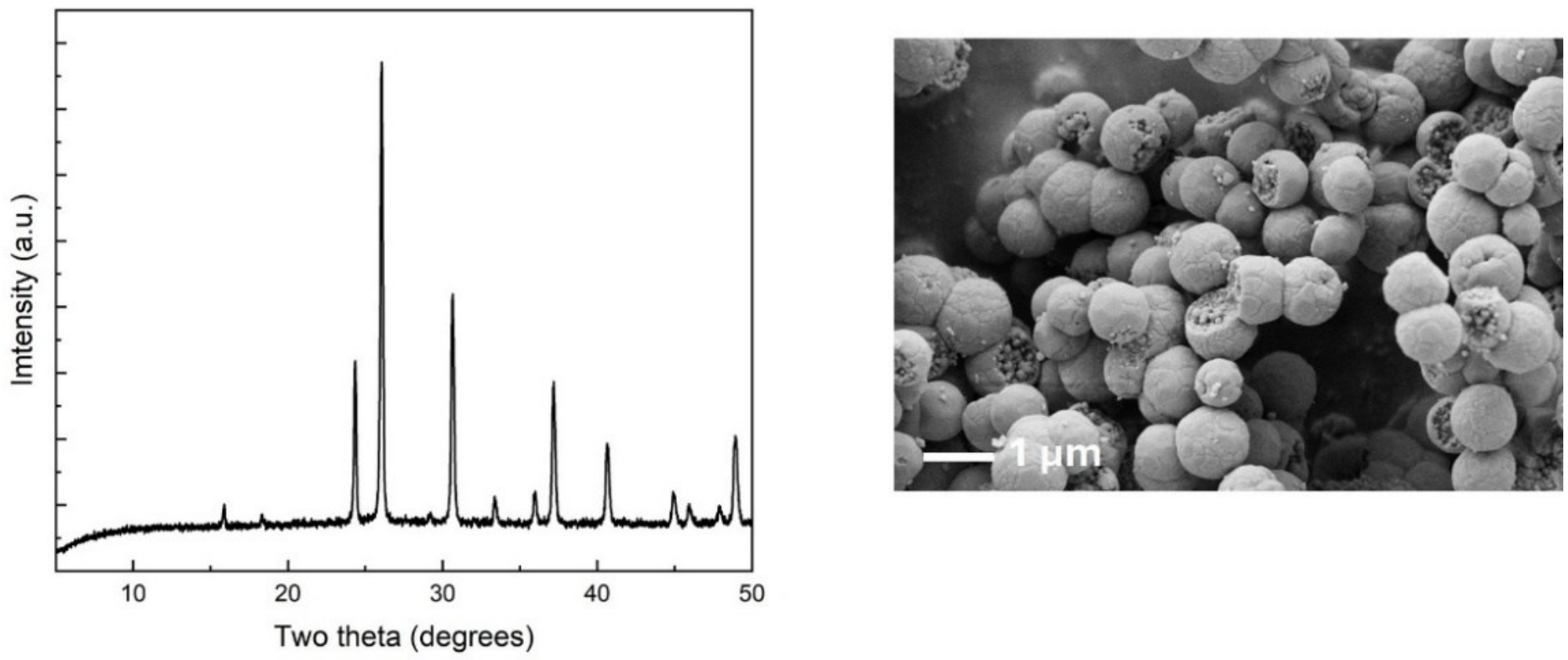

Figure S5. XRD pattern (left) and SEM image (right) showing the ANA zeolite made with 1.18 g CsOH for 4 days.

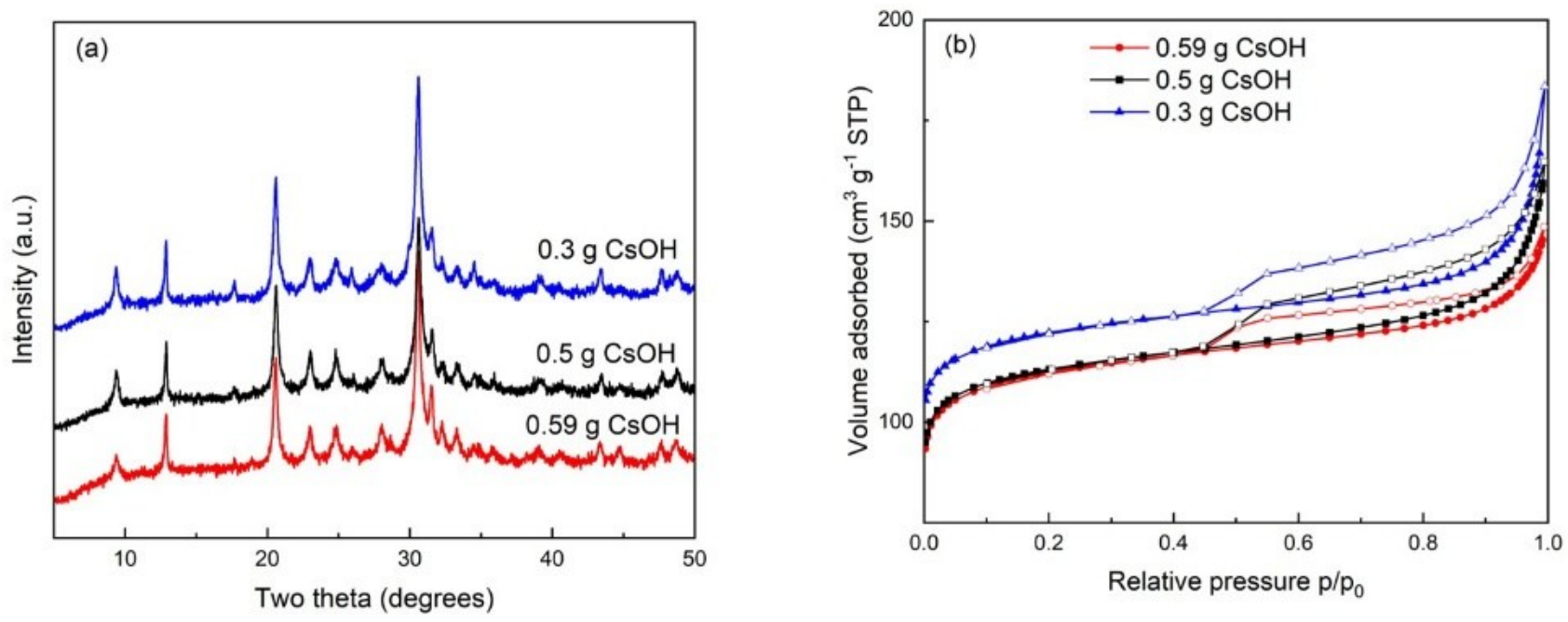

Figure S6. (a) XRD patterns and (b) nitrogen adsorption isotherms of Cs-CHA samples prepared with different amounts of CsOH.

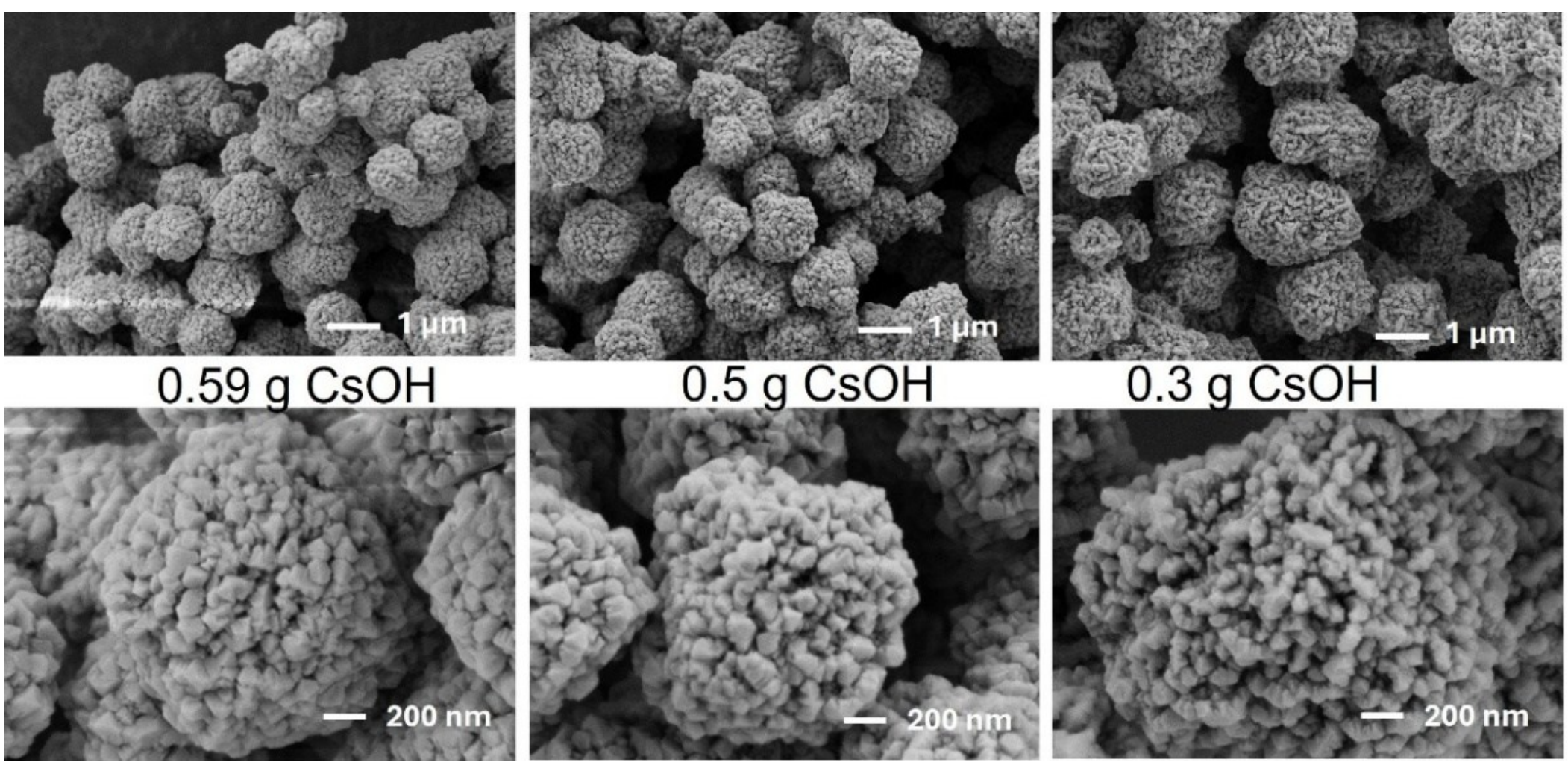

Figure S7. SEM images of Cs-CHA samples prepared with different CsOH content.

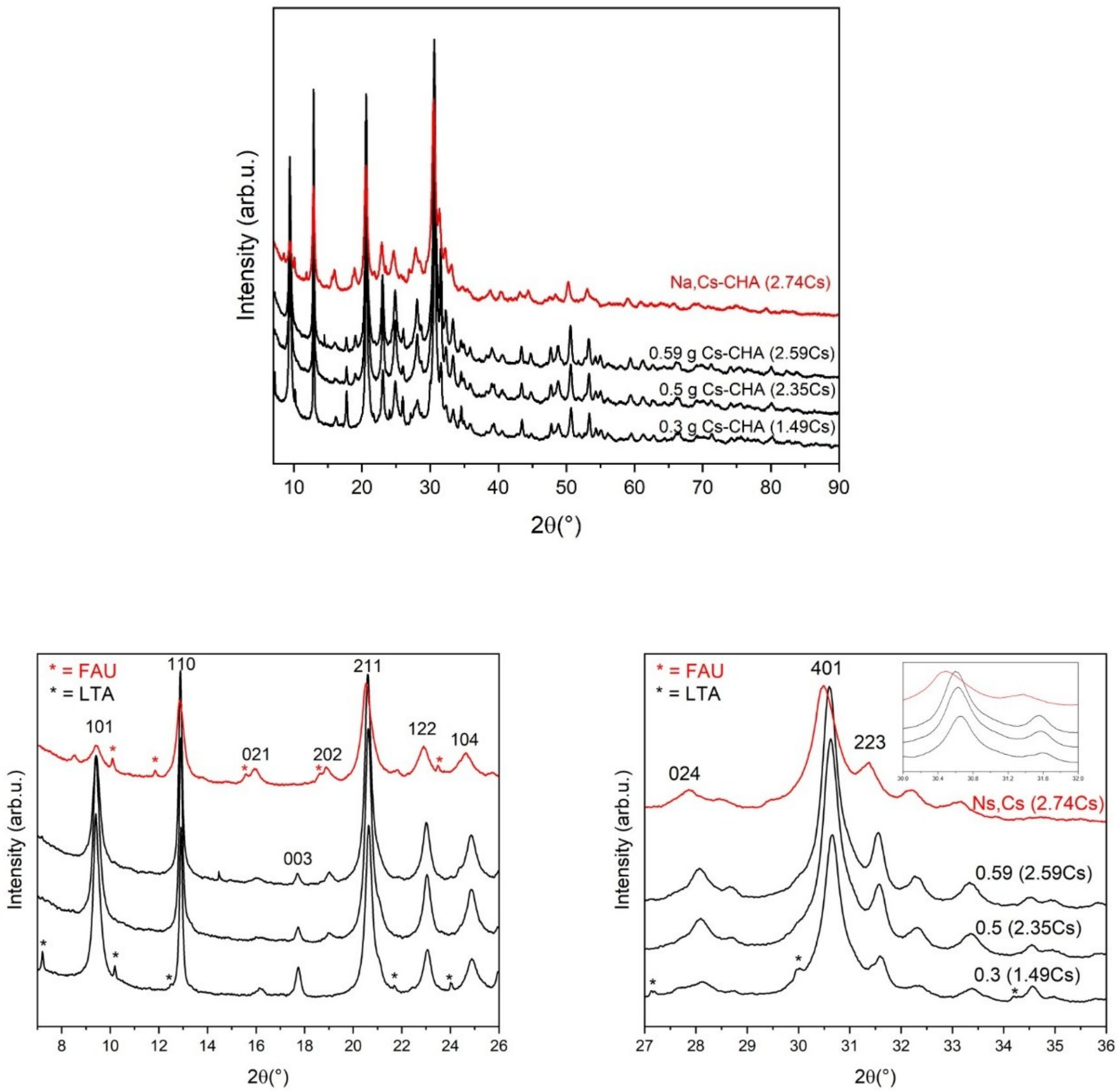

Figure S8. PXRD collected for Le Bail refinement of Cs-CHA samples prepared with different Cs content and the Na,Cs-CHA sample. The numbers in brackets indicate the Cs content per unit cell.

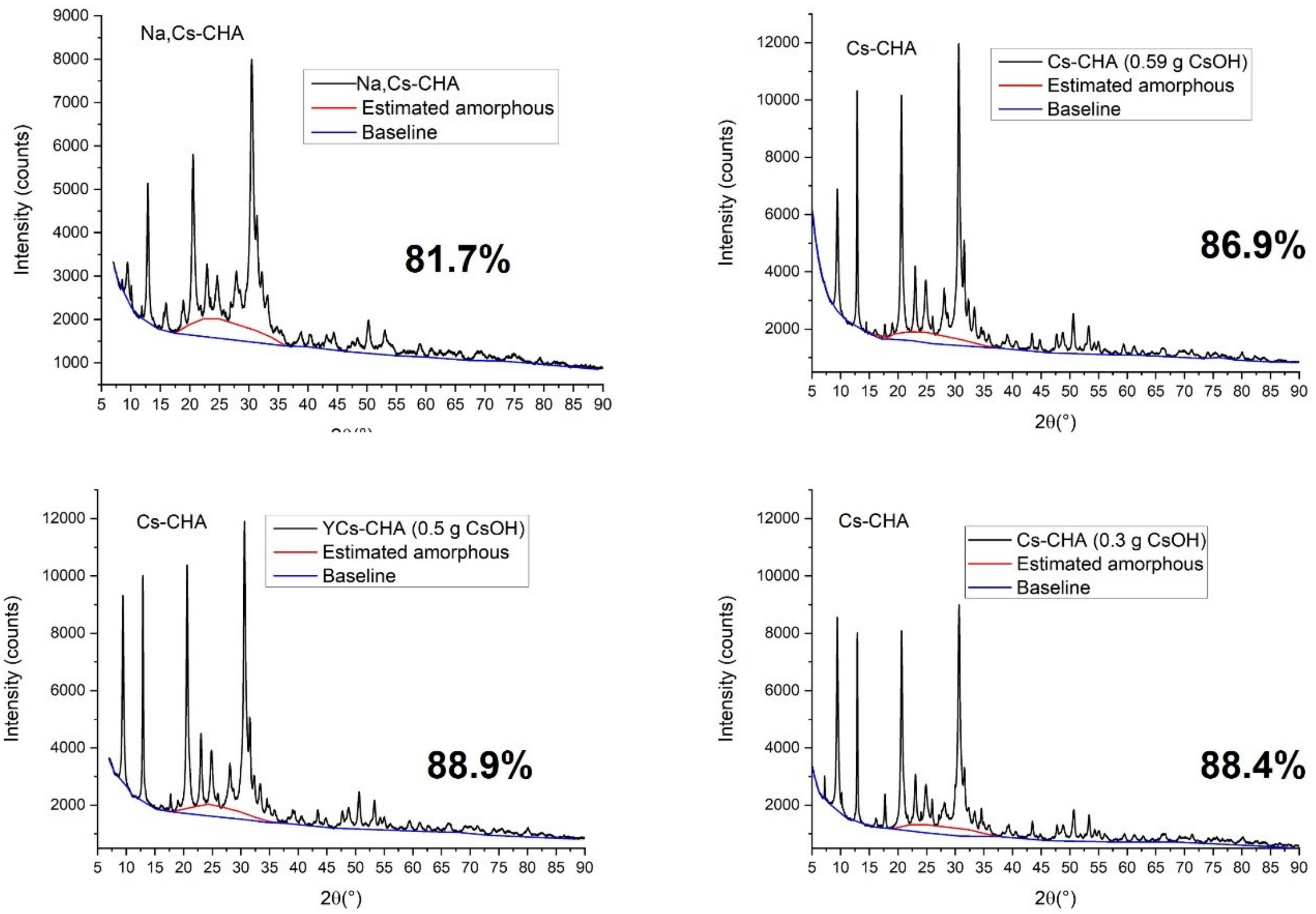

Figure S9. Relative crystallinity estimates derived from integrated diffraction peak intensities for the Ns,Cs-CHA and Cs-CHA samples.

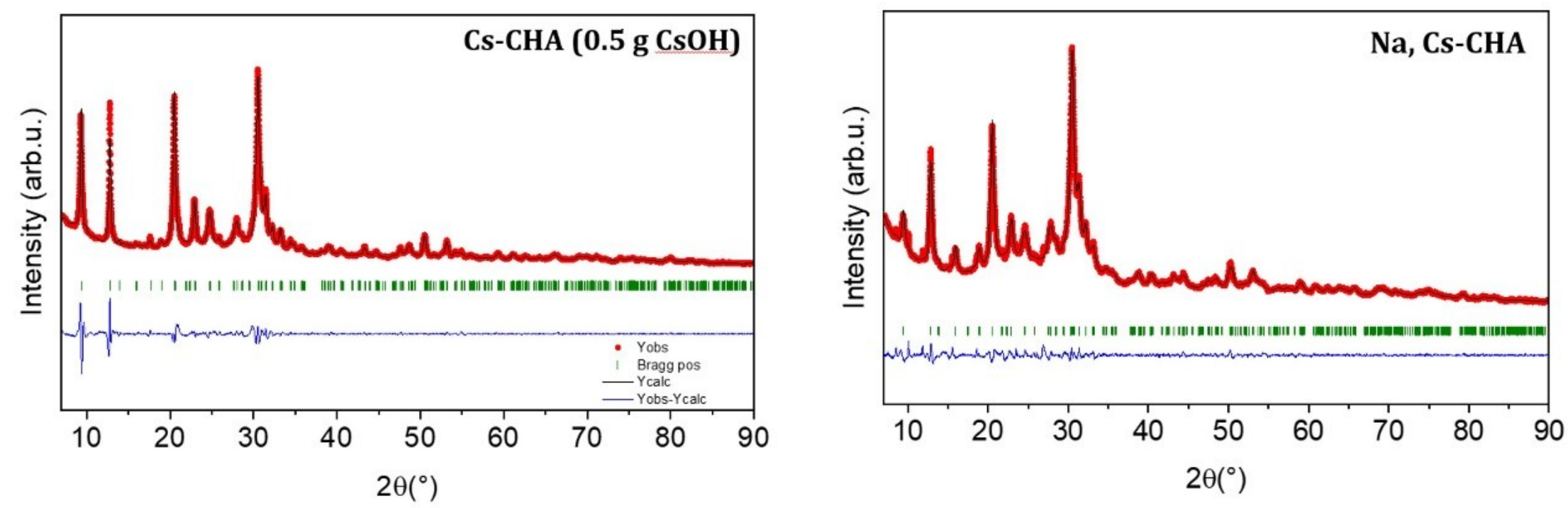

Figure S10. Le Bail refinement of Cs-CHA and Na,Cs-CHA samples. The observed, calculated, and difference profiles are shown as red, black, and blue lines, respectively. Vertical tick marks indicate the Bragg reflection positions.

Table S4. Summary of Le Bail refinement data.

Compounds	Space group	a (Å)	c (Å)	V (Å^3)	Rp (%)	Rwp (%)	Chi^2
**Na, Cs-CHA**	*R*-3*m* (*H*)	13.8082(4)	15.2138(7)	2512.11(15)	1.75	2.38	0.96
**0.59 g Cs-CHA (2.59Cs)**	*R*-3*m* (*H*)	13.7587(2)	15.0556(6)	2468.27(10)	3.13	4.83	4.17
**0.5 g Cs-CHA (2.35Cs)**	*R*-3*m* (*H*)	13.7417(2)	15.0495(5)	2461.12(9)	3.08	4.89	4.29
**0.3 g Cs-CHA (1.49Cs)**	*R*-3*m* (*H*)	13.7169(4)	15.0337(6)	2449.47(15)	4.54	7.21	6.71

Table S5. Chemical composition (mol. ratios) and textural characteristics of Na,Cs-CHA samples of higher Si/Al ratios (in bold) compared to the reference Na,Cs-CHA and the 0.5 g Cs-CHA samples.

Sample	CsOH, g	NaOH, g	Si/Al	Cs/Al	Na/Al	S_{BET} (m^2 g^{-1})	V_{micro} (cm^3 g^{-1})	V_{total} (cm^3 g^{-1})
Na,Cs-CHA, 1 day	0.5	0.91	1.67	0.20	0.91	113.7	0.028	0.09
**Na,Cs-CHA, 1 day**	**0.7**	**0.35**	**1.95**	**0.39**	**0.77**	**218.9**	**0.060**	**0.18**
**Na,Cs-CHA, 2 days**	**0.7**	**0.20**	**2.06**	**0.40**	**0.75**	**316.1**	**0.103**	**0.26**
Cs-CHA, 7 days (upscaled)	0.5	-	2.19	0.21	0.88	368.4	0.142	0.25

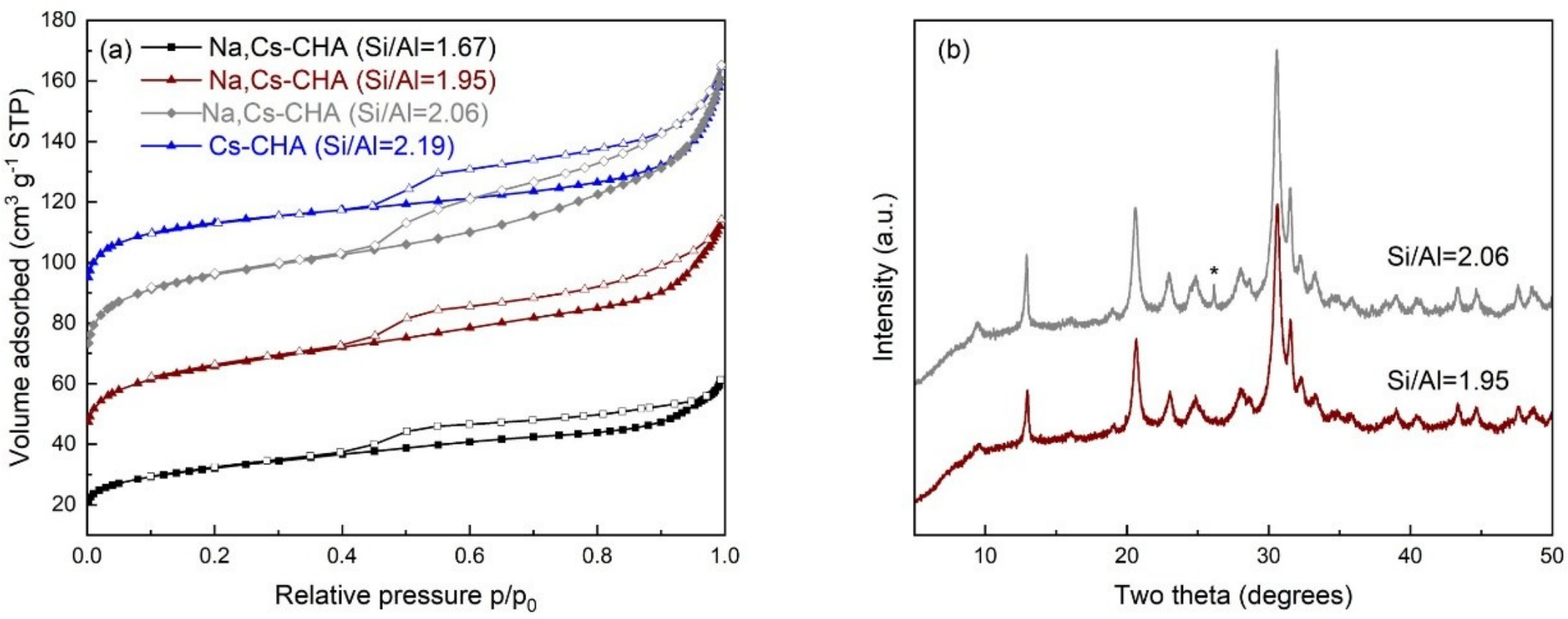

Figure S11. (a) **N**itrogen adsorption isotherms of the Na,Cs-CHA and Cs-CHA samples from described in Table S5 and (b) XRD patterns of the Na,Cs-CHA samples with a higher Si/Al ratio; * indicates ANA impurities.
